# Coexisting Thoracoabdominal Aortic Tortuosity With Multifocal Atherosclerosis and Bilateral Renal Venous Tributaries Variations: A Cadaveric Case Report

**DOI:** 10.7759/cureus.116269

**Published:** 2026-09-15

**Authors:** Gonzalo Del Rio, Andrea Fabregas, Azarel Rosa, Maria Santa Maria, Angelisa Franceschini, Bárbara Riestra-Candelaria

**Affiliations:** 1 Department of Anatomy and Cell Biology, Universidad Central del Caribe, School of Medicine, Bayamón, PRI; 2 Department of Pathology, Universidad Central del Caribe, School of Medicine, Bayamón, PRI

**Keywords:** aortic tortuosity, atherosclerosis, renal vein tributaries variation, surgical anatomy, thoracoabdominal aorta, vascular anatomy

## Abstract

Aortic tortuosity is a documented anatomical finding associated with alterations in vascular structure, arterial wall modifications, and age-related degenerative alterations. Variations in the anatomy of renal venous tributaries are also frequent and may have important implications for retroperitoneal surgery, transplantation, and vascular interventions. During a routine gross anatomy lab cadaveric dissection of an adult female of unknown age, diffuse thoracoabdominal aortic tortuosity, atherosclerosis, and bilateral renal venous tributary variations were identified. The aorta followed a markedly tortuous course involving both the thoracic and abdominal segments and demonstrated a tortuosity index of 1.29. Examination of the opened vessel revealed multifocal atherosclerotic changes in the thoracic and abdominal aorta. Grade III atherosclerosis, characterized by calcified plaques and focal intimal ulcerations, was observed within the aortic arch. Bilateral multiple hilar venous tributaries converged into the main renal veins, with a posteriorly positioned tributary contributing to an atypical hilar arrangement on the left side. This case documents the coexistence of diffuse thoracoabdominal aortic tortuosity, atherosclerotic disease, and variations in bilateral renal venous tributaries in a cadaver. These findings highlight the value of cadaveric dissection in identifying clinically relevant vascular anatomy and underscore the importance of recognizing complex aortic and renal venous configurations during anatomical education, radiologic interpretation, surgical planning, and endovascular procedures.

## Introduction

Clinical anatomy education often focuses on the textbook description of vascular structures. However, cadaveric dissection reveals that anatomical variation is common and can influence disease presentation, imaging, and surgical planning. Unusual vascular architecture in the thorax or retroperitoneum may increase operative risk or alter hemodynamic stresses. Age-related elongation and tortuosity of the thoracic and abdominal aorta have been associated with progressive degeneration of elastic fibers, increased collagen deposition, and reduced arterial compliance. Loss of elastin reduces the aorta's ability to recoil, whereas increased collagen stiffness and mechanical stress promote elongation (the “creep phenomenon”) and angulation of the vessel, leading to alterations in normal vascular geometry [1-4]. This process is often accentuated by chronic hypertension and atherosclerosis.

Tortuous aortas are generally asymptomatic but may act as a biomarker of vascular degeneration. Reported causes of aortic tortuosity include advancing age, genetic connective tissue disorders (Marfan, Loeys-Dietz, or arterial tortuosity syndromes), and long-standing hypertension [2-4]. Extreme tortuosity can complicate endovascular procedures because twists increase the risk of graft migration, endoleak, or vessel injury. Knowledge of normal and variant aortic geometry is therefore essential for thoracic endovascular aortic repair or valve replacement. Likewise, variations in the renal veins are not uncommon and should be anticipated during retroperitoneal surgery. Anatomical studies report that the right renal vein is short and drains directly into the inferior vena cava, whereas the left vein is longer and therefore preferred for living‑donor nephrectomy [5]. Variations in renal venous anatomy may include accessory veins, multiple hilar venous tributaries, or anomalous drainage patterns of adjacent veins, underscoring the need for careful preoperative imaging and anatomical awareness [5]. Although thoracoabdominal aortic tortuosity and renal venous variations have each been described independently, the coexistence of marked aortic tortuosity with bilateral multiple renal venous tributary variations in a single cadaver is uncommon and provides an opportunity to examine anatomical relationships that may have clinical and surgical implications.

## Case presentation

As part of routine educational dissection activities, this anatomical study was performed on an embalmed cadaver provided by the Junta de Donaciones Anatómicas de Puerto Rico to the Gross Anatomy laboratory at Universidad Central del Caribe. Standard thoracic and abdominal dissections were carried out following institutional protocols. The thoracic cavity was opened by removing the anterior rib cage to expose the mediastinum, and the abdominal viscera were mobilized to visualize the retroperitoneal vasculature.

Once the thoracoabdominal aorta was isolated, its morphology and anatomical course were observed and documented. The aorta was observed to pass through the aortic hiatus of the diaphragm (conventionally located at the level of the twelfth thoracic vertebra (T12)) before continuing into the abdominal cavity. The total length of the aorta was measured directly along its centerline using a flexible measuring tape. The straight-line distance between the aortic arch (just after the left subclavian artery branch) and the abdominal aortic bifurcation (at the beginning point of the common iliac arteries bifurcation) was also measured. A tortuosity index (TI) was calculated as the ratio of total aortic length (L) to the straight-line distance (D) between the aortic arch and the aortic bifurcation (TI = L/D).

Then the thoracoabdominal aorta was longitudinally opened along its course to allow direct examination of the luminal surface and arterial wall. A pathologist participating in the study evaluated the presence, distribution, and severity of atherosclerotic changes, including intimal plaques, calcifications, and ulcerations. Findings were documented and correlated with gross anatomical observations.

For evaluation of the renal vasculature, the kidneys were dissected in situ following removal of the perirenal fat. Arterial and venous structures were traced from their origins at the abdominal aorta and inferior vena cava to the renal hila. Special attention was paid to documenting the configuration of the renal venous tributaries and their anatomical relationships within the renal hilum. Findings were documented photographically and compared with standard published anatomical descriptions.

No personal identifiers were associated with the cadaver, and all procedures complied with institutional guidelines governing the use of donated human body remains for education and research.

Aortic tortuosity

The thoracoabdominal aorta demonstrated a markedly tortuous course rather than the relatively straight descending trajectory typically described in anatomical texts. Immediately distal to the origin of the left subclavian artery, the descending thoracic aorta deviated prominently toward the left and followed a posterior course relative to the vertebral column. As it approached the thoracoabdominal transition, the vessel curved toward the right while remaining anterior to the vertebral column. Inferior to the origins of the renal arteries, the abdominal aorta again deviated toward the left before continuing to the aortic bifurcation. These sequential deviations produced a diffuse tortuous configuration involving both the thoracic and abdominal segments of the aorta (Figure 1). No gross aneurysmal dilatation was identified.

**Figure 1:**
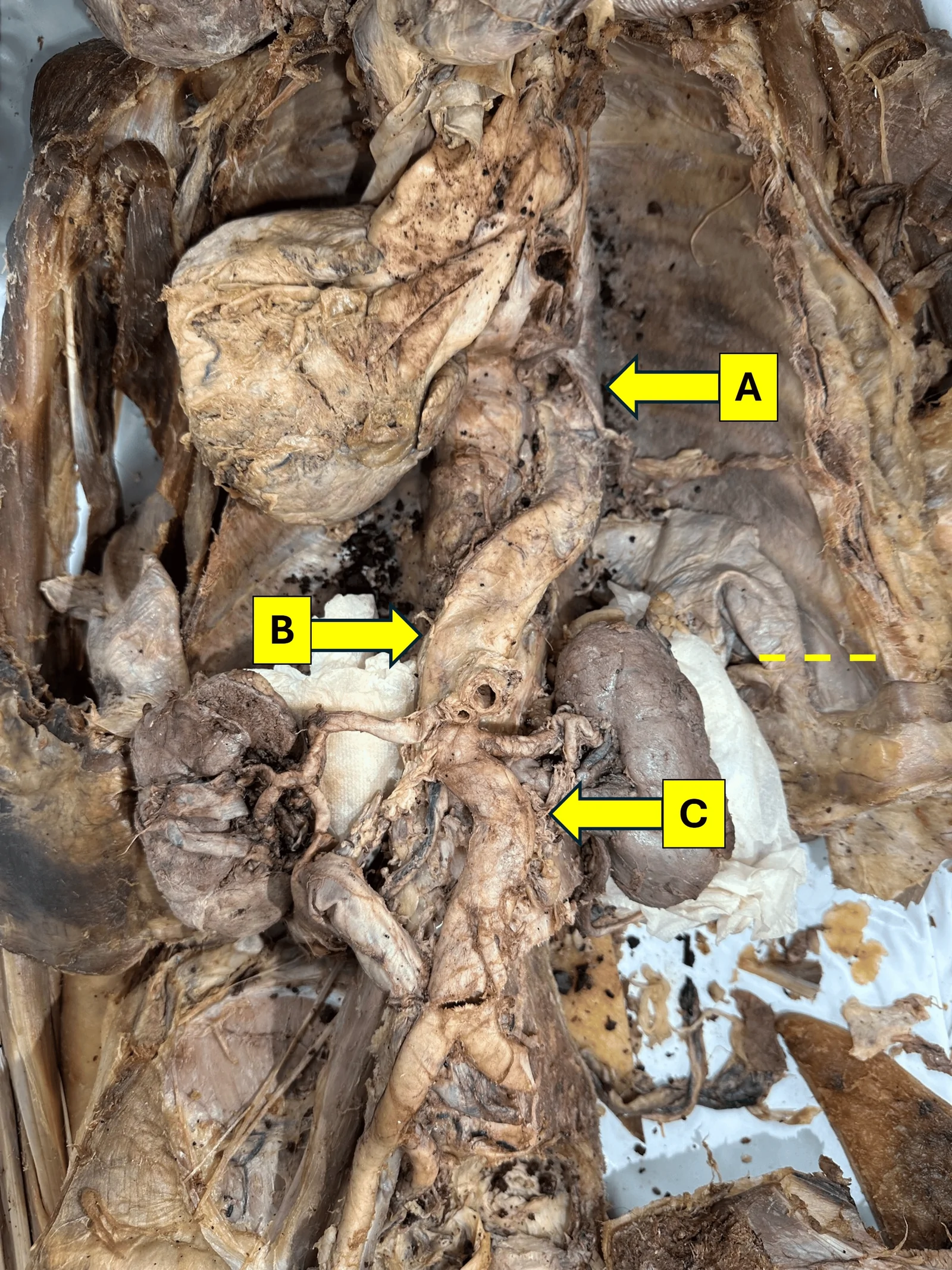
Course of the thoracoabdominal aorta demonstrating: (A) leftward and posterior deviation distal to the left subclavian artery, (B) rightward curvature at the thoracoabdominal transition anterior to the vertebral column, (C) leftward deviation inferior to the renal arteries. The dashed line indicates the inferior extent of the diaphragm. The heart was manually repositioned to the right to facilitate visualization of the aorta.

Examination of the opened aorta revealed mild-to-moderate atherosclerotic changes involving both the thoracic and abdominal segments. The most advanced lesions were identified in the aortic arch, where grade III atherosclerosis was present, characterized by prominent calcified atherosclerotic plaques and focal intimal ulcerations (Figure 2).

**Figure 2:**
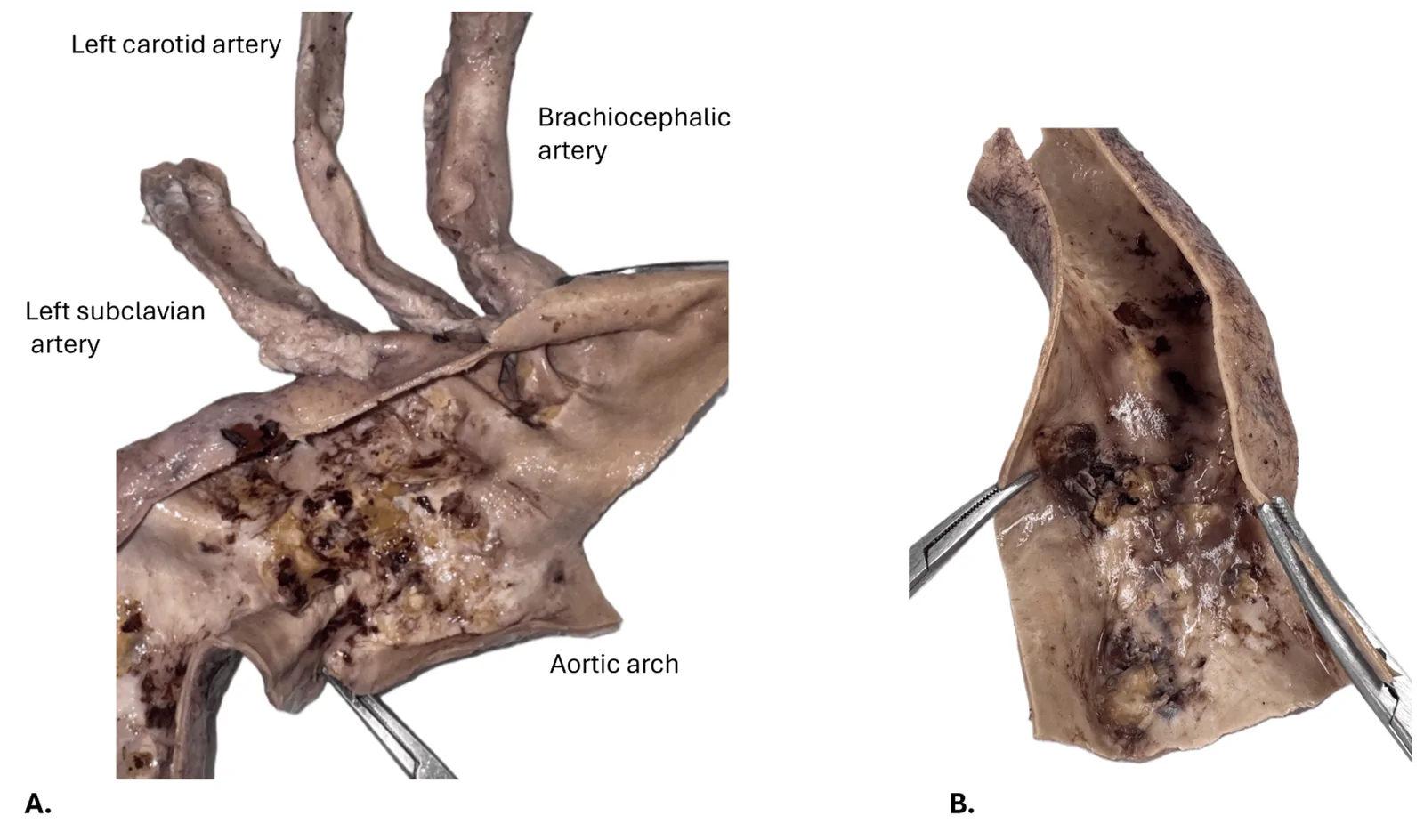
Internal view of the thoracoabdominal aorta. (A) Aortic arch demonstrating advanced grade III atherosclerosis with calcified plaques and focal intimal ulcerations. (B) Thoracic aorta demonstrating mild-to-moderate atherosclerotic changes.

The thoracoabdominal aorta measured 46.5 cm in total length. The straight-line distance between the aortic arch and the aortic bifurcation measured 36.0 cm, yielding a tortuosity index of 1.29 (Figure 3).

**Figure 3:**
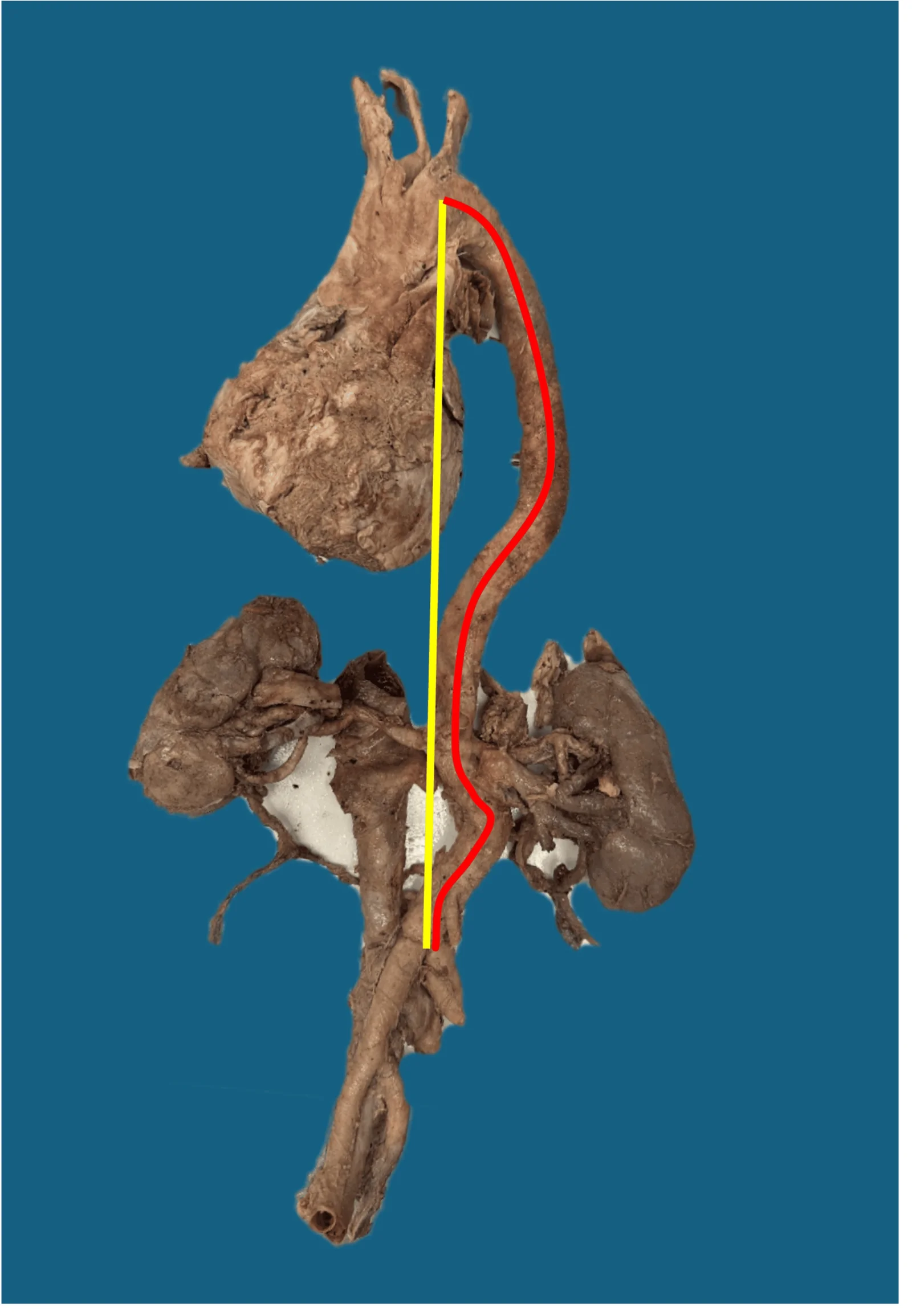
Gross anterior view of the thoracoabdominal aorta demonstrating diffuse tortuosity and elongation. The red line indicates the measured centerline length of the thoracoabdominal aorta, which was 46.5 cm. The yellow line indicates the straight-line distance between the aortic arch and the aortic bifurcation, which measured 36.0 cm. The calculated tortuosity index was 1.29.

Renal venous variations

Bilateral renal venous variations were identified during retroperitoneal dissection. Multiple venous tributaries emerged from each renal hilum and converged to form the main renal vein before draining into the inferior vena cava (Figure 4).

**Figure 4:**
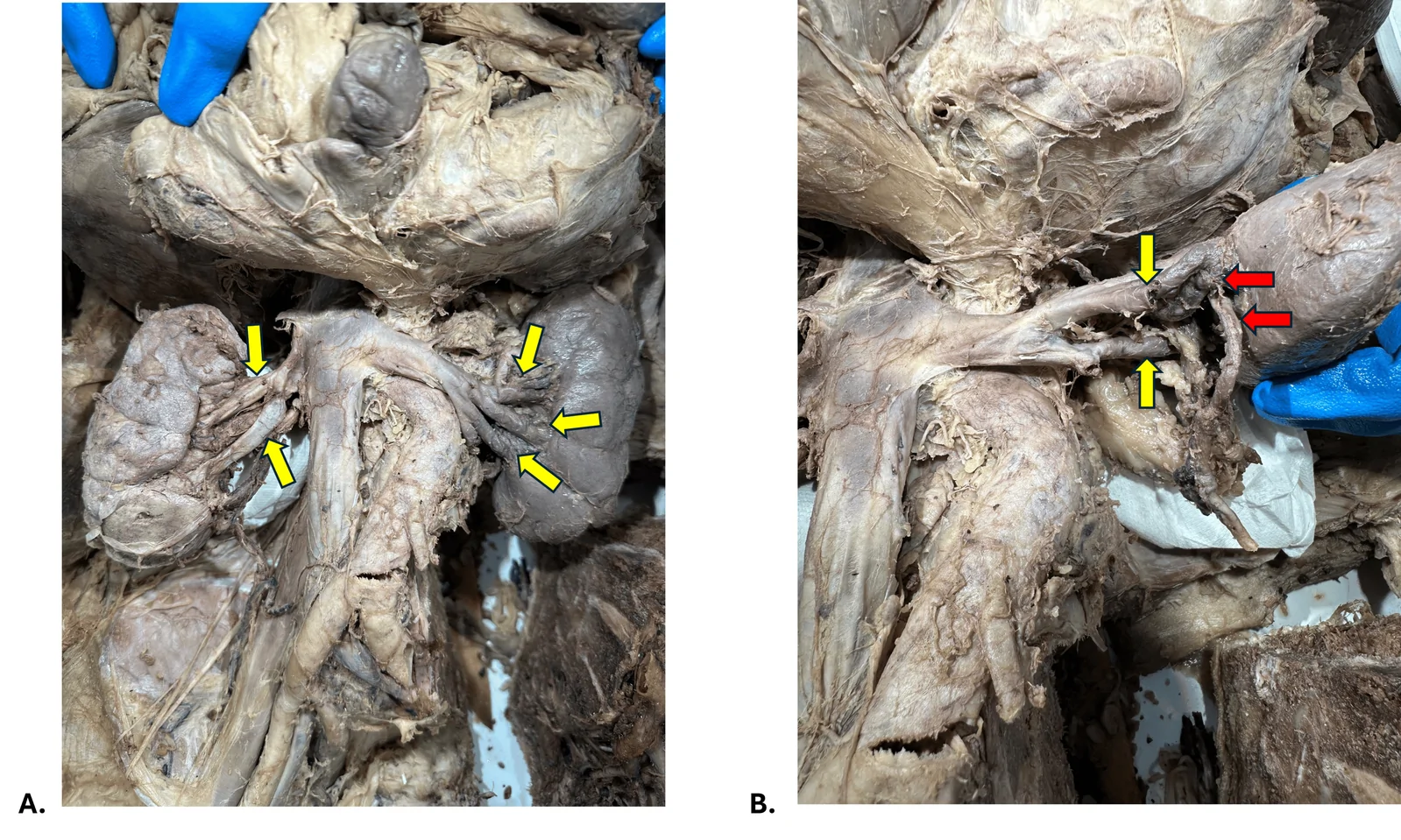
Renal venous tributary variations. (A) Bilateral renal venous tributaries (yellow arrows) converging toward the main renal veins. (B) Left renal hilum demonstrating a posteriorly positioned venous tributary relative to the remaining hilar venous tributaries, with a renal artery branching and the ureter positioned between venous tributaries (red arrows).

On the right side, the renal venous tributaries maintained the typical anterior relationship to the renal arterial branches within the renal hilum, with a superior and an inferior tributary converging into the right renal vein. In contrast, the left renal hilum demonstrated a more complex arrangement, in which three venous tributaries converged into an anterior renal vein, and two venous tributaries converged into a posterior renal vein, which subsequently joined to form a single left renal vein. The gonadal vein drained into the inferior tributary of the anterior left renal vein. Although most venous tributaries were located anterior to the renal arterial branches, one venous tributary coursed posterior to the remaining venous channels. As a result, the renal vascular structures and the ureter were positioned between venous tributaries, resulting in an atypical hilar configuration compared with the classical anterior-to-posterior arrangement. No evidence of duplicated renal veins with distinct caval terminations was observed.

## Discussion

Thoracoabdominal aortic tortuosity has been increasingly recognized as a manifestation of age-related vascular remodeling and altered arterial biomechanics. Previous studies have demonstrated that with advancing age, the abdominal aorta may undergo progressive elongation and increased tortuosity, altering its normal anatomical course and its spatial relationship with adjacent vascular structures. These changes may be particularly significant in individuals with variant renal venous anatomy, where displacement or distortion of the aorta can contribute to compression or compromise of the renal veins. Age-related changes in the aortic wall play an important role in this process. Over time, degeneration and fragmentation of elastic fibers, together with increased collagen deposition and fibrosis, reduce the elasticity and compliance of the vessel wall [1-4]. These structural changes promote arterial stiffening, elongation, and tortuosity. As the aorta becomes increasingly tortuous, the resulting alteration in vascular geometry may modify the normal relationship between the aorta and the renal veins, potentially increasing the risk of venous compression in susceptible anatomical variants.

Although the age and medical history of the present cadaver were unknown, the observed anatomical configuration is consistent with patterns previously reported to be associated with vascular aging. The identification of multifocal atherosclerotic disease in the thoracic and abdominal aorta, together with advanced grade III atherosclerosis characterized by calcified plaques and intimal ulcerations within the aortic arch, provides additional morphological evidence of chronic arterial remodeling that may have contributed to the marked thoracoabdominal tortuosity observed in this specimen.

The tortuosity index of 1.29 quantitatively supports the gross anatomical impression of a markedly elongated vessel. Unlike isolated focal curvature, the present specimen demonstrated sequential deviations involving both the thoracic and abdominal segments of the aorta. The vessel first deviated toward the left and posteriorly after the origin of the left subclavian artery, then curved toward the right at the thoracoabdominal transition, and finally returned toward the left inferior to the renal arteries. This multidirectional configuration highlights the three-dimensional nature of aortic tortuosity and emphasizes the value of direct anatomical observation in understanding vascular morphology. Although the aorta was observed passing through the aortic hiatus of the diaphragm, the vertebral levels corresponding to the individual curvatures were not specifically documented during dissection, thereby limiting precise correlation with vertebral levels.

Tortuous aortas are frequently asymptomatic but may have important procedural implications. Previous investigations have shown that excessive aortic tortuosity may complicate endovascular interventions by increasing device manipulation, limiting catheter advancement, and reducing procedural stability [2-4]. In addition, abdominal aortic tortuosity identified on computed tomography has been associated with an increased risk of complications during transfemoral transcatheter aortic valve replacement, underscoring the clinical relevance of preoperative assessment of aortic geometry [6]. Recognition of these vascular variations on computed tomography angiography, magnetic resonance angiography, and other cross-sectional imaging studies is important for accurate anatomical interpretation and procedural planning. The present case, therefore, underscores the importance of recognizing complex aortic configurations during surgical planning and image interpretation.

The relationship between aortic tortuosity and anatomical variations of the renal veins is an important consideration in vascular anatomy, radiology, and surgery. The renal venous findings observed in this specimen differed from the classical description of a single renal vein emerging from the renal hilum and draining directly into the inferior vena cava [5]. Rather than duplicated renal veins or separate caval terminations, the variation consisted of multiple hilar venous tributaries converging into the main renal vein bilaterally. Particularly notable was the presence of a posteriorly positioned venous tributary within the left renal hilum, creating a multilayered arrangement in which vascular structures and the ureter were situated between venous channels.

Variations of the renal venous system are clinically important because they may increase the complexity of retroperitoneal surgery, renal transplantation, partial nephrectomy, and endovascular procedures [5]. Awareness of atypical hilar venous branching patterns may reduce the risk of inadvertent vascular injury and facilitate surgical dissection. From an educational perspective, this case also shows how cadaveric dissection continues to provide valuable insight into anatomical variations that textbook descriptions alone may not fully capture.

## Conclusions

This cadaveric case report documents the coexistence of diffuse thoracoabdominal aortic tortuosity and bilateral renal venous branching variations in an adult female cadaver. The aorta demonstrated a markedly elongated and tortuous course, with a tortuosity index of 1.29 and sequential deviations involving both the thoracic and abdominal segments. Pathologic examination additionally revealed multifocal atherosclerotic changes in the thoracic and abdominal aorta, with advanced grade III atherosclerosis, calcification, and focal intimal ulceration within the aortic arch. In addition, multiple renal venous tributaries bilaterally converged to form the main renal veins, with an unusually positioned posterior tributary contributing to a complex hilar arrangement on the left.

These findings highlight the diversity of vascular anatomy and emphasize the continued value of cadaveric dissection in identifying anatomical variations that may not be fully appreciated through standard descriptions. Awareness of complex aortic configurations and atypical renal venous patterns may facilitate surgical planning, improve anatomical interpretation, and reduce the risk of inadvertent vascular injury during retroperitoneal and endovascular procedures. Surgeons, radiologists, and clinicians should therefore consider aortic tortuosity and renal venous anomalies when evaluating patients, planning interventions, and teaching future health professionals.
